# Effects of COVID-19-related stress and fear on depression in schizophrenia patients and the general population

**DOI:** 10.1038/s41537-022-00213-3

**Published:** 2022-03-05

**Authors:** Yu-Ri Lee, Young-Chul Chung, Jung Jin Kim, Shi Hyun Kang, Bong Ju Lee, Seung-Hwan Lee, Jonghun Lee, Ha-Ran Jung, Jinhee Hyun, Min Jhon, Ju-Wan Kim, Seunghyong Ryu, Ju-Yeon Lee, Jae-Min Kim, Sung-Wan Kim

**Affiliations:** 1 Department of Social Welfare, Nambu University, Gwangju, Korea; 2 Department of Psychiatry, Chonbuk National University Medical School, Jeonju, Korea; 3 Department of Psychiatry, The Catholic University of Korea, Seoul St. Mary’s Hospital, Seoul, Korea; 4 Department of Social Psychiatry and Rehabilitation, National Center for Mental Health, Seoul, Korea; 5 Department of Psychiatry, Inje University Haeundae Paik Hospital, Inje University College of Medicine, Busan, Korea; 6 Department of Psychiatry, Inje University College of Medicine, Goyang, Korea; 7 Department of Psychiatry, Catholic University of Daegu, College of Medicine, Daegu, Korea; 8 Daegu Metropolitan Mental Health and Welfare Center, Daegu, Korea; 9 Department of Psychiatry, Naju National Hospital, Naju, Korea; 10 Department of Social Welfare, College of Social Sciences, Daegu University, Gyeongsan, Korea; 11 Department of Psychiatry, Chonnam National University Medical School, Gwangju, Korea; 12 Mindlink, Gwangju Bukgu Community Mental Health Center, Gwangju, Korea

**Keywords:** Psychosis, Schizophrenia

## Abstract

This study compared the coronavirus disease 2019 (COVID-19)-related stress, fear of infection, loneliness, and depression between patients with schizophrenia and the general population. A face-to-face survey was administered to 1340 patients with a schizophrenia spectrum disorder and online survey of the general population (*n* = 2000) was conducted. The information gathered included the level of COVID-19-related stress, fear of infection, the Patient Health Questionnaire-9 score, and the three-item UCLA Loneliness Scale score. Structural equation modeling revealed a significant effect of fear of COVID-19 infection on depression among the general population and on loneliness among patients with schizophrenia. Loneliness experienced during COVID-19 exacerbated depression in both groups. In the COVID-19-related stress–loneliness–depression pathway, the partial mediating effect of loneliness was significant in both groups. Conversely, in the COVID-19-related fear–loneliness–depression pathway, the full mediating effect of loneliness was only significant in patients with schizophrenia. In conclusion, the loneliness associated with COVID-19-related stress and fear of infection was an important factor influencing depression, and the impact was greater in patients with schizophrenia compared with the general population. Thus, different mental health intervention plans are needed for patients with schizophrenia during the COVID-19 pandemic. During the long-lasting COVID-19 pandemic, social support and provision of mental health services to prevent loneliness and consequent depression are required in patients with schizophrenia.

## Introduction

Infectious disease outbreaks have caused psychosocial distress and various emotional problems associated with changes in people’s daily lives and the fear of infection^1–3^. The coronavirus disease 2019 (COVID-19) pandemic, which has caused social isolation, economic stress, fear of infection, and constraints on people’s daily lives, has worsened mental health and increased the risk of depression in the general population^4–7^. Furthermore, the COVID-19 outbreak is a long-lasting, worldwide pandemic that has created a social disaster. The COVID-19 pandemic has infiltrated every aspect of our daily lives and has drastically changed our social and healthcare systems^8^. Individuals with a mental illness are more vulnerable to such a disaster, as are children, youth, older adults, pregnant women, people with physical disabilities, residents of communal living facilities, and foreign residents^9^.

People with schizophrenia may be more susceptible to transmission of COVID-19 and have higher mortality rates^10–14^. Patients with schizophrenia may be more vulnerable to stress due to the COVID-19 pandemic, as with other disasters. Basically, patients with schizophrenia who exhibit functional impairments and negative symptoms also tend to have limited social interactions. In addition, during the COVID-19 pandemic, social distancing policies and fear of infection may further restrict social activity and disconnect people from social support^8^. In particular, the COVID-19 pandemic has restricted access to medical resources and community mental health services and has thus increased loneliness and the risk of exacerbation^15,16^. Although the need for services for socially and mentally vulnerable populations including patients with schizophrenia has increased, social and mental health supports have been limited during the pandemic^17^.

Previous studies on mental health issues, including psychosocial stress and depression associated with COVID-19, have been conducted mainly in the general population. Few studies have examined depression in patients with schizophrenia, although they are among those considered vulnerable to COVID-19 pandemic issues^10^. Thus, differences in psychosocial stress and mental health patterns associated with COVID-19 between people with mental illness and the general population must be examined, and appropriate intervention strategies must be implemented.

The hypotheses of our study were as follows. First, the levels of psychological distress and depression are likely high in schizophrenia patients during the COVID-19 pandemic. Second, the pathway causing depression may differ in patients with schizophrenia compared with the general population. To address these hypotheses, we employed structural equation modeling (SEM) and latent mean analysis of major psychologic variables, including the stress associated with the COVID-19 outbreak, fear of infection, loneliness, and depression, in patients with schizophrenia compared with the general population. Such multidimensional comparative analyses in patients with schizophrenia and the general population may provide basic data to establish specialized intervention strategies for people with schizophrenia.

## Results

### Participant sociodemographic characteristics

A total of 1340 patients with a schizophrenia spectrum disorder and 2000 members of the general population participated in this study. Table 1 displays the sociodemographic characteristics of the participants. There was no difference in age or sex between the patients and general population. However, patients with schizophrenia were significantly more likely to be single, unemployed, or less educated.

**Table 1:** Sociodemographic characteristics of participants.

		Patients with schizophrenia (*n* = 1340)	General population (*n* = 2000)	
Gender	male	653 (48.8%)	1000 (50.0%)	χ^2^ = 0.532
	female	687 (51.2%)	1000 (50.0%)	*p* = 0.466
Education level	≤12 years	596 (44.5%)	346 (17.3%)	χ^2^ = 562.215
	>12 years	744 (55.5%)	1654 (82.7%)	*p* < 0.001
Employment status	employed	333 (24.9%)	1407 (70.4%)	χ^2^ = 280.273
	unemployed	1007 (75.1%)	593 (29.6%)	*p* < 0.001
Marital status	married	404 (30.1%)	1032 (51.6%)	χ^2^ = 280.912
	single	936 (69.9%)	968 (48.4%)	*p* < 0.001
Age		40.1 ± 12.2	39.3 ± 11.6	*p* = 0.060

### Descriptive statistics

In SEM, the assumption of multivariate normal distributions cannot be satisfied if the measured variables do not form a normal distribution. This leads to a distorted estimate, and an accurate statistical verification cannot be performed. Considering the conditions for normality (skewness <2, kurtosis <7)^18^ with SEM, the skewness and kurtosis of the variables selected in this study satisfied the necessary conditions to apply SEM (Suppl. Table 1).

### Comparative analysis using latent mean analysis

The results of the invariance analysis conducted for the latent mean analysis satisfied the requirements for configural invariance, metrics invariance, and scalar invariance. First, the configural invariance was verified to be at a level that was satisfactory for both the patients with schizophrenia and the general population (Table 2). Second, verification of the metrics invariance indicated that the difference in χ^2^ values between the metrics invariance model and the configural invariance model in the two comparison groups was not statistically significant at the 5% level. Additionally, as the TLI, CFI, and RMSEA fit indices did not decrease, metrics invariance was established (Table 2).

**Table 2:** Goodness-of-fit Indices of invariance tests between the two groups.

Patients with schizophrenia-General population	Model Fit					
	χ^2^	df	*p*	TLI	CFI	RMSEA
Configural invariance	3827.927	538	0.000	0.914	0.923	0.061
Metrics invariance	3855.707	559	0.000	0.920	0.922	0.060
Scalar invariance	3885.814	580	0.000	0.923	0.920	0.059
Factor variance invariance	3893.612	584	0.000	0.922	0.916	0.059

*df* degree of freedom, *CFI* Comparative fit index, *TLI* Tucker-Lewis Index, *RMSEA* Root Mean Square Error of Approximation.

Third, verification of the scalar invariance showed that the difference in χ^2^ values between the metrics invariance model and the scalar invariance in the two comparison groups was not statistically significant at the 5% level. Additionally, as the TLI, CFI, and RMSEA fit indices did not decrease, scalar invariance was established (Table 2). Hence, the observed differences in the latent variable means can be considered to reflect actual differences between the groups (Table 2).

As the variance invariance was satisfied (Table 2), the effect size of the latent mean difference was analyzed using the common standard deviation for each comparative group model. The results are shown in Table 3. Estimation of the latent mean for schizophrenia patients, with the latent mean of the general population fixed to 0, revealed that the levels of fear and stress associated with COVID-19 infection was significantly lower in patients with schizophrenia than in the general population. Furthermore, the levels of loneliness and depression were significantly higher in patients with schizophrenia than in the general population. The effect size of the latent mean difference was medium for the fear of COVID-19 infection, large for stress related to COVID-19, medium for loneliness, and small for depression (Table 3).

**Table 3:** Latent mean analysis between the two groups.

Category	Latent means difference/Significant probability		Cohen’s d
	General population (*n* = 2000)	Patients with schizophrenia (*n* = 1340)	
COVID-19 Fear	0	−0.506**	0.60
COVID-19 Stress	0	−0.793***	0.97
Loneliness	0	0.371**	0.57
Depression	0	0.264*	0.29

*p < 0.05, **p < 0.01, ***p < 0.001.

$${{{\mathrm{Cohen}}}}^\prime {{{\mathrm{s}}}}\;{{{\mathrm{d}}}} = \frac{{{{{\mathrm{group}}}}1\;{{{\mathrm{latent}}}}\;{{{\mathrm{mean}}}}\,-\, {{{\mathrm{group}}}}2\;{{{\mathrm{latent}}}}\;{{{\mathrm{mean}}}}}}{{\sqrt {{{{\mathrm{common}}}}\;{{{\mathrm{factor}}}}\;{{{\mathrm{variance}}}}\;{{{\mathrm{of}}}}\;{{{\mathrm{two}}}}\;{{{\mathrm{groups}}}}} }}$$

### Comparative analysis via verification by SEM

**Fig. 1:**
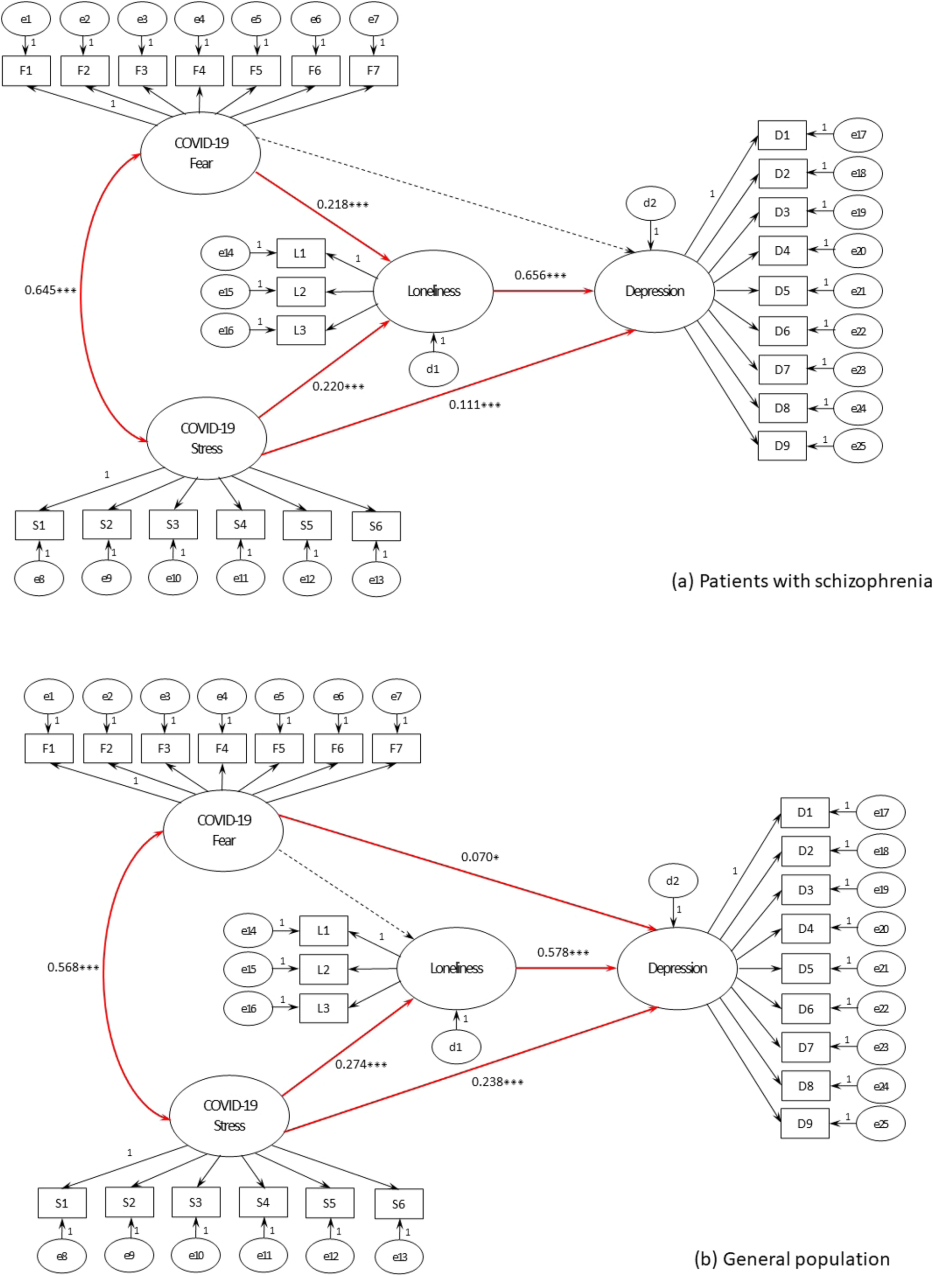
Structural equation model linking COVID-19-related fear and stress, loneliness, and depression in patients with schizophrenia and the general population. **a** Pathways and estimates in patients with schizophrenia. **b** Pathways and estimates in the general population. Given values are standardized estimates. *p < 0.05, **p < 0.01, ***p < 0.001. Red solid lines are statistically significant, and black dotted lines are not statistically significant.

In this study, the model fit was verified using CFI, TLI, and RMSEA, which are not sensitive to sample size, consider the simplicity of the model, and have established criteria for evaluating the fit^19^. The fit of the study model was satisfactory for all indices except χ^2^ (Fig. 1, Table 4)^20^. The results for the direct effects were as follows. First, the effect of fear of COVID-19 infection on depression was not significant in patients with schizophrenia (β = 0.657, *p* > 0.05) but statistically significant in the general population (β = 2.621, *p* < 0.05). The effect of fear of COVID-19 infection on loneliness was statistically significant in patients with schizophrenia (β = 5.382, *p* < 0.001) but not in the general population (β = 1.086, *p* > 0.05). The effects of COVID-19-related stress on depression and loneliness were statistically significant for both patients with schizophrenia (β = 3.281, *p* < 0.001 and β = 5.262, *p* < 0.001, respectively) and the general population (β = 7.810, *p* < 0.001 and β = 7.644, *p* < 0.001, respectively). Similarly, the effect of loneliness on depression was statistically significant in both patients with schizophrenia (β = 16.992, *p* < 0.001) and the general population (β = 20.045, *p* < 0.001). Compared with the other variables, loneliness had the greatest effect on depression (Table 4). Additionally, controlling for the sociodemographic characteristics showed a significant effect on the major variables in several pathways (Table 4).

**Table 4:** Estimates of the study model and the mediating effects.

		Patients with schizophrenia				General population			
Path		B	β	S.E.	C.R.	B	β	S.E.	C.R.
COVID-19Fear → Depression		0.014	0.021	0.021	0.657	0.050	0.070	0.019	2.621*
COVID-19Fear → Loneliness		0.100	0.218	0.019	5.382***	0.021	0.035	0.019	1.086
Loneliness → Depression		1.037	0.656	0.061	16.992***	0.703	0.578	0.035	20.045***
COVID-19Stress → Depression		0.091	0.111	0.028	3.281***	0.194	0.238	0.025	7.810***
COVID-19Stress → Loneliness		0.115	0.220	0.022	5.262***	0.184	0.274	0.024	7.644***
COVID-19Fear ↔ COVID-19Stress		0.586	0.645	0.036	16.123***	0.359	0.568	0.022	16.458***
Control variables	Gender → COVID-19Fear	−0.125	−0.081	0.123	−1.016	−0.136	−0.105	0.128	−1.062
	Gender → COVID-19Stress	−0.139	−0.090	0.094	−1.477	−0.155	−0.114	0.141	−1.099
	Gender → Loneliness	−0.094	−0.073	0.065	−1.446	−0.068	−0.060	0.072	−0.094
	Gender → Depression	−0.075	−0.069	0.093	−0.806	−0.175	−0.118	0.103	−1.697
	Age → COVID-19Fear	0.479	0.299	0.172	2.785*	0.434	0.295	0.168	2.583*
	Age → COVID-19Stress	0.095	0.077	0.104	0.911	0.369	0.264	0.153	2.410*
	Age → Loneliness	0.088	0.071	0.112	0.784	−0.428	−0.310	0.143	−2.993**
	Age → Depression	0.081	0.065	0.129	0.626	−0.625	−0.523	0.074	−8.445***
	Marital status → COVID-19Fear	0.293	0.172	0.438	0.668	0.372	0.199	0.185	2.011*
	Marital status → COVID-19Stress	0.186	0.119	0.252	0.737	0.389	0.208	0.210	1.852*
	Marital status → Loneliness	−0.363	−0.302	0.059	−6.151***	−0.475	−0.364	0.055	−8.636***
	Marital status → Depression	0.299	0.187	0.333	0.896	−0.267	−0.228	0.053	−5.038***
	Educational level → COVID-19Fear	0.105	0.029	1.112	0.093	0.093	0.030	0.082	1.132
	Educational level → COVID-19Stress	0.081	0.027	0.192	0.421	0.078	0.023	0.135	0.576
	Educational level → Loneliness	0.202	0.082	0.378	0.534	0.036	0.011	0.054	0.666
	Educational level → Depression	−0.309	−0.179	0.063	−4.903***	−0.401	−0.294	0.056	−7.160***
	Job → COVID-19Fear	−0.292	−0.093	0.518	−0.563	−0.412	−0.373	0.269	−1.531
	Job → COVID-19Stress	0.384	−0.099	0.579	−0.663	−0.623	−0.588	0.184	−3.386*
	Job → Loneliness	−0.517	−0.468	0.078	−6.626***	−0.861	−0.724	0.083	−10.373***
	Job → Depression	−0.831	−0.678	0.104	−7.990***	−0.921	−0.870	0.088	−10.466***
Mediating effect		β	S.E.	95%CI	P	β	S.E.	95%CI	P
COVID-19Fear → Loneliness→ Depression		0.143	0.028	(0.085, 0.197)	0.002	0.020	0.020	(−0.023, 0.056)	0.420
COVID-19Stress → Loneliness→ Depression		0.144	0.029	(0.093, 0.208)	0.001	0.158	0.021	(0.121, 0.201)	0.001
Goodness of fit	χ^2^	1667.870 (*p* = 0.000, df = 269)				2160.057 (*p* = 0.000, df = 269)			
	TLI	0.923				0.905			
	CFI	0.931				0.915			
	RMSEA	0.062				0.059			

*p < 0.05, **p < 0.01, ***p < 0.001

*B* unstandardized estimate, *β* standardized estimate, *S.E.* standard error, *C.R.* critical ratio

95%CI 95% Confidence Interval Bias-corrected, *df* degree of freedom, *CFI* Comparative fit index, *TLI* Tucker-Lewis Index, *RMSEA* Root Mean Square Error of Approximation

Sociodemographic characteristics were controlled when analyzing the direct and indirect effects.

The indirect effect of a variable is the portion of the total effect that is mediated by other variables in the model. A comparative analysis of the indirect effects in the fear of COVID-19 infection–loneliness–depression pathway revealed a significant mediating effect of loneliness in patients with schizophrenia (β = 0.143, *p* = 0.002); however, the effect was not significant in the general population (β = 0.020, *p* = 0.420). Comparative analysis of the indirect effects in the stress–loneliness–depression pathway revealed a significant partial mediating effect in both patients with schizophrenia (β = 0.144, *p* = 0.001) and participants from the general population (β = 0.158, *p* = 0.001) (Table 4).

## Discussion

Patients with schizophrenia are a mentally vulnerable population during the COVID-19 pandemic. However, research focusing on mental health issues associated with COVID-19 in patients with schizophrenia are insufficient. This is the study to investigate differential pathways of the effects of COVID-19-related stress and fear of infection on depression between patients with schizophrenia and the general population. In this study, patients with schizophrenia showed lower levels of fear of infection and COVID-19-related stress but higher levels of loneliness and depression compared with the general population. In the COVID-19-related stress–loneliness–depression pathway, the partial mediating effect of loneliness was significant both in patients with schizophrenia and in the general population. Conversely, in the fear of infection–loneliness–depression pathway, the mediating effect of loneliness was only significant in patients with schizophrenia. We believe that our study results, derived from multidimensional comparative analysis, offer timely suggestions regarding the need for social and mental health support to prevent loneliness and depression in patients with schizophrenia.

The latent mean analysis showed that the stress associated with COVID-19 and the fear of infection were lower in patients with schizophrenia than in the general population. The effect size of the latent mean difference was large for COVID-19-related stress, suggesting that the continuing global COVID-19 pandemic has caused great stress in the general population. Lifestyle changes due to the COVID-19 pandemic, such as decreased social interaction and economic activity, might be relatively greater in the general population than in patients with schizophrenia. Furthermore, patients with schizophrenia may be more preoccupied with serious intrinsic issues that existed before the COVID-19 pandemic than with COVID-19-related stress^21^. In addition, patients with schizophrenia may attribute their fear and stress to causes other than COVID-19.

The prevalence of depression in the general population has reportedly increased considerably in Asian and Western countries during the pandemic^22–24^. However, in the present study, patients with schizophrenia were shown to be more vulnerable to loneliness and depression during the pandemic than the general population. Previous studies reported potentially high levels of loneliness and depression among patients with schizophrenia during the COVID-19 pandemic^17^. Moreover, the results of our study showed larger differences in the latent means for loneliness and depression compared with the general population. Depression in patients with schizophrenia is related to poorer clinical outcome, low resilience, high suicidality, and reduced subjective quality of life^25,26^. The COVID-19 pandemic has reduced access to medical resources and weakened community mental health services. Social distancing and restrictions on mental health support might easily have increased loneliness and depression among patients with schizophrenia. Therefore, public mental health services for this vulnerable population should be strengthened. Non-contact mental health service strategies for patients with schizophrenia are also required to minimize service gaps during long-term pandemic situations^27^. Mental health clinicians should also consider depression and loneliness as essential targets in case management.

In this study, the effect of the fear of COVID-19 infection on depression was not significant in patients with schizophrenia, but it was significant in the general population. In contrast, the effect of the fear of COVID-19 infection on loneliness was significant in patients with schizophrenia but not in the general population. Previous studies of the general population showed results similar to our study, with a positive association between the fear of COVID-19 infection and depression^5–8,28^. However, in our study, the fear of COVID-19 infection was not the cause of depression in patients with schizophrenia. Previous studies reported that during infectious outbreaks, vulnerable groups such as people with disabilities or mental illnesses show limited attention to information or guidelines associated with the infectious disaster due to their functional disabilities^9,10,12^. Moreover, the high level of intrinsic fear associated with the psychopathology of schizophrenia might influence this relatively low fear of infection. Although fear of infection did not directly increase the level of depression, it indirectly exacerbated depression via the mediating effect of loneliness, which was increased by fear of infection in patients with schizophrenia. The questionnaire on fear of infection included worries regarding familial infection, quarantine, and restrictions on hospital visits^29^. Thus, fear of infection in patients with schizophrenia might be strongly associated with loneliness, which can lead to depression. Social disconnection and a shortage of mental health support during the COVID-19 pandemic may increase loneliness in patients with schizophrenia more easily than the general population. The sense of loneliness associated with social isolation during the COVID-19 pandemic was found to have a large impact on exacerbating depression in both patients with schizophrenia and the general public^16,17,23^. Therefore, the maintenance of social and emotional interactions is required to reduce the impact of loneliness on depression, despite the long-term and inevitable social distancing policies during the pandemic.

Our study applied the major demographic variables as control variables in the study model. Older age affected the fear of COVID-19 infection in both populations, whereas younger age affected loneliness and depression in the general population. The fact that COVID-19 is significantly more fatal to the older population may increase the fear of infection in older populations^30^. However, in terms of emotional distress and other sequalae of COVID-19, younger people may be a more vulnerable population during the COVID-19 pandemic^31,32^. For the group of patients with schizophrenia, marital status and occupation affected the sense of loneliness, and occupation and education level affected the sense of depression. These results suggest the need for interventions to reduce the fear of COVID-19 infection among older patients with schizophrenia and to reduce the sense of loneliness and depression among unmarried, unemployed, or less educated patients with schizophrenia.

This study has a few limitations. First, the analysis of the mediating effects and the latent means was focused solely on loneliness as a psychological trait that acts as a factor influencing depression during the COVID-19 pandemic. However, it would be important to expand the spectrum of possibly influential factors, including overall health status and the severity of the psychopathology. Second, we should consider the differing effects of a paper-and-pencil and online surveys. The answers to some delicate questions might differ between the face-to-face and online surveys. In future, latent mean and path analyses are required using the same survey method for both groups. Third, while the COVID-19 pandemic strongly influenced Korean society, most people in the Korean region did not undergo lockdown. Therefore, our results should be carefully adjusted before they are applied to other countries. Finally, this was a cross-sectional study conducted during the first year of the COVID-19 pandemic. It may be that patients with schizophrenia are more lonely and depressed than the general population even in the absence of a pandemic. A longitudinal study that investigates causal relationships and the long-term impacts of the pandemic on mental health is required. A multivariate latent growth model should be used to estimate changes in variables over time and to verify relationships among such changes. Despite these limitations, our study has significant implications in that it provides a theoretical basis for further study and for the establishment of mental health intervention strategies for patients with schizophrenia in the context of insufficient studies of this vulnerable population during the COVID-19 pandemic.

In conclusion, loneliness associated with COVID-19-related stress and fear of infection was an important factor influencing depression, and the impact was greater in patients with schizophrenia compared with the general population. During this long-lasting COVID-19 pandemic, social support and the provision of mental health service to prevent loneliness and consequent depression are required in patients with schizophrenia.

## Methods

### Research participants

This study is part of a mental health survey on the psychosocial effects of COVID-19 in patients with schizophrenia and the general population in Korea. The survey comprised two parts for both patients with psychosis and the general population. The patient inclusion criteria were: (1) age 19–65 years, (2) current treatment at a community mental health center or psychiatric outpatient clinic, (3) diagnosis (by the treating psychiatrist) of a schizophrenia-spectrum disorder based on DSM-5 criteria^33^, and (4) competence in terms of questionnaire completion and provision of informed consent. The exclusion criteria were: (1) a significant, clinically unstable, and uncontrolled physical or mental illness; and/or (2) an inability to understand the investigation or to provide informed consent given a severe impairment evident on reality testing. Most data collections were conducted via a paper-and-pencil survey. Some participants (<2%) responded to the survey via an online platform. An online survey was deemed preferable to a face-to-face survey given the need for social distancing. However, we surveyed patients face-to-face because we believed that they were less comfortable with smartphones than the general population. This study was conducted between April and July 2020, i.e., during the COVID-19 pandemic. The study purpose and process were presented to the participants, and their consent was obtained before proceeding with the study.

Second, the survey for the general population was conducted online. Details of the data collection method for the general population were described previously^29^. Briefly, we conducted an anonymous online survey of residents aged 19–65 years from three metropolitan areas according to the prevalence of COVID-19 using a quota sampling method considering age and sex. The online survey was conducted via a service provider (Macromill Embrain) in which all subjects were panelists. The first data collection period was between April and May 2020 and included 1,500 people. The second online survey was conducted in July to match 500 people in terms of age and sex. Electronic informed consent was obtained from each participant prior to starting the investigation. The study was approved by the Chonnam National University Hospital Institutional Review Board (CNUH-2020-092) and by each institute for the survey for patients with psychosis.

### Measures

The measures and data used in this study were the participants’ sociodemographic data, including sex, age, marital status (married vs. single), education level, and employment status (regular or temporarily employed vs. not).

Depression was measured using the Patient Health Questionnaire-9^34,35^. Cronbach’s α for this scale was 0.897. Loneliness, as an index of psychological status, was measured using the UCLA Loneliness Scale, which comprises three items rated on a 3-point Likert scale from “Rarely” (1 point) to “Often” (3 points); the maximum possible score is 9, with higher scores signifying a stronger sense of loneliness^36,37^. Cronbach’s α for this scale was 0.837.

A questionnaire developed and validated by the authors was used to identify fear of infection and stress associated with COVID-19^29^. The psychometric properties of this questionnaire were presented in a previous paper^29^. The questionnaire comprised seven questions pertaining to the fear of infection and six to the stress associated with COVID-19. All questions were scored using a 5-point Likert scale (1, strongly disagree; 2, disagree; 3, neutral; 4, agree; 5, strongly agree). The Cronbach’s α values for this questionnaire were 0.838 and 0.921 for the fear of infection and stress components, respectively.

### Statistical analysis

The SPSS 20.0 and AMOS 20.0 programs were used for data analysis. First, Cronbach’s α was calculated to measure the reliability of each scale, frequency and normality analyses were conducted, and means and standard deviations were derived.

Second, a latent mean analysis was performed to compare mean latent variables with controlled measurement error and to identify differences between the groups. In contrast to ANOVA, which compares mean values directly, latent mean analysis has the advantage of considering the measurement error between variables. To perform latent mean analysis, the configural invariance, metrics invariance, and scalar invariance of the model must be established^38,39^. The test for configural invariance was conducted by setting a base model that enabled correlations among all of the latent variables with free parameter estimation. The test for metrics invariance was conducted by calculating the χ^2^ values, degrees of freedom, and fit indices such as the Tucker–Lewis Index (TLI), comparative fit index (CFI), and root mean square error of approximation (RMSEA). This test was performed to identify any significant differences between the two groups for the metrics invariance model with invariance constraints and the configural invariance model with no constraints. The test for scalar invariance was performed by comparing the χ^2^ values, degrees of freedom, and fit indices, such as the TLI, CFI, and RMSEA between the metrics and scalar invariance models.

Once the configural invariance, metrics invariance, and scalar invariance were satisfied, the latent mean analysis of COVID-19-related stress, fear of COVID-19 infection, loneliness, and depression was performed. As the mean values of the factors cannot be compared directly in latent mean analysis, it is only possible to estimate the latent mean of the other groups, while the latent mean of the reference group is fixed at 0^40,41^. Cohen’s effect size was used to verify the relative effects of the latent means estimated in the latent mean analysis process^42^. The common standard deviation for the effect size calculation was calculated by securing the factor variance invariance. Statistical significance was verified by performing χ^2^ difference tests between the scalar invariance model and the models with invariance constraints applied between factors^38^. Third, SEM was performed for comparative analysis of the direct and indirect effects. Goodness-of-fit indices, such as χ^2^, CFI, TLI, and RMSEA were used to evaluate the model fit. The significance of mediating effects was verified using the bootstrapping method.

### Reporting Summary

Further information on research design is available in the Nature Research Reporting Summary linked to this article.

## Supplementary information

Supplementary Table 1

REPORTING SUMMARY

## Data Availability

The data that provide the findings of this study are available from the corresponding author upon reasonable request.
