# Supplementary material for: Effects of COVID-19-related stress and fear on depression in schizophrenia patients and the general population
Source: Schizophrenia (Heidelb). 2022 Mar 5;8(1):15. doi: 10.1038/s41537-022-00213-3 (PMC8897617; doi:10.1038/s41537-022-00213-3)
Supplement: Supplementary file 1 — Supplementary Table 1 [file 41537_2022_213_MOESM1_ESM.pdf]

Supplementary Table 1. Descriptive statistics of variables

	Minimum	Maximum	Mean	S.D.	Skewness	Kurtosis
**Patients with schizophrenia**						
COVID-19 Stress	1.00	5.00	2.4366	.90552	.223	-.401
Stress1	1.00	5.00	2.3582	1.14585	.459	-.735
Stress2	1.00	5.00	2.1119	1.03674	.738	-.028
Stress3	1.00	5.00	2.6657	1.28768	.269	-.988
Stress4	1.00	5.00	2.3619	1.16632	.500	-.558
Stress5	1.00	5.00	2.7978	1.26308	.073	-1.048
Stress5	1.00	5.00	2.3239	1.15796	.566	-.518
COVID-19 Fear	1.00	5.00	3.0512	1.09384	-.263	-.732
Fear1	1.00	5.00	3.0963	1.27438	-.202	-1.027
Fear2	1.00	5.00	3.2903	1.31405	-.394	-.964
Fear3	1.00	5.00	2.9209	1.33478	.011	-1.163
Fear4	1.00	5.00	2.9276	1.26124	-.027	-1.051
Fear5	1.00	5.00	2.9866	1.31168	-.079	-1.125
Fear6	1.00	5.00	3.3209	1.31913	-.402	-.930
Fear7	1.00	5.00	2.8157	1.25634	.091	-.992
Loneliness	1.00	3.00	1.8975	.64099	.272	-.945
Loneliness1	1.00	3.00	2.0082	.69507	-.011	-.928
Loneliness2	1.00	3.00	1.8687	.76073	.225	-1.244
Loneliness3	1.00	3.00	1.8157	.76395	.325	-1.225
Depression	.00	3.00	.8195	.74507	.978	.254
Depression1	.00	3.00	.9119	.98214	.940	-.110
Depression2	.00	3.00	.8821	.96595	.940	-.086
Depression3	.00	3.00	1.1157	1.09758	.605	-.960
Depression4	.00	3.00	1.0194	1.00614	.741	-.519
Depression5	.00	3.00	.8813	.97930	.913	-.209
Depression6	.00	3.00	.7881	.98450	1.088	.068
Depression7	.00	3.00	.6910	.97239	1.284	.491
Depression8	.00	3.00	.6343	.90010	1.345	.856
Depression9	.00	3.00	.4963	.86029	1.781	2.245
**General population**						
	Minimum	Maximum	Mean	S.D.	Skewness	Kurtosis
COVID-19 Stress	1.00	5.00	3.0406	.77998	-.084	-.119
Stress1	1.00	5.00	2.9145	1.16913	-.014	-.950
Stress2	1.00	5.00	2.2600	1.08027	.588	-.392
Stress3	1.00	5.00	3.4915	1.16345	-.484	-.607
Stress4	1.00	5.00	2.9555	1.20509	-.036	-.942
Stress5	1.00	5.00	3.6960	1.01936	-.687	-.028
Stress5	1.00	5.00	2.9260	1.09047	-.008	-.718
COVID-19 Fear	1.00	5.00	3.8131	.78600	-.666	.394
Fear1	1.00	5.00	3.8000	1.02349	-.716	.001
Fear2	1.00	5.00	4.1255	.91004	-1.067	.996
Fear3	1.00	5.00	3.5315	1.20030	-.438	-.797
Fear4	1.00	5.00	3.7830	.99670	-.708	.052
Fear5	1.00	5.00	3.7325	1.10660	-.710	-.230
Fear6	1.00	5.00	4.3885	.79805	-1.527	2.831
Fear7	1.00	5.00	3.3310	1.13185	-.275	-.759
Loneliness	1.00	3.00	1.7465	.60634	.497	-.649

Loneliness1	1.00	3.00	1.9085	.70169	.129	-.968
Loneliness2	1.00	3.00	1.6800	.70416	.541	-.864
Loneliness3	1.00	3.00	1.6510	.68807	.581	-.774
Depression	.00	3.00	.6704	.58714	1.169	1.210
Depression1	.00	3.00	.8230	.85851	.970	.410
Depression2	.00	3.00	.7490	.81384	1.035	.697
Depression3	.00	3.00	.9025	.96460	.869	-.227
Depression4	.00	3.00	1.1045	.89944	.689	-.154
Depression5	.00	3.00	.7560	.84488	1.004	.400
Depression6	.00	3.00	.6040	.82615	1.369	1.263
Depression7	.00	3.00	.4400	.72087	1.690	2.427
Depression8	.00	3.00	.3175	.64335	2.187	4.562
Depression9	.00	3.00	.3370	.68313	2.209	4.559

S.D.: standard deviation
