# Supplementary material for: Effects of COVID-19-related stress and fear on depression in schizophrenia patients and the general population
Source: Schizophrenia (Heidelb). 2022 Mar 5;8(1):15. doi: 10.1038/s41537-022-00213-3 (PMC8897617; doi:10.1038/s41537-022-00213-3)
Supplement: Supplementary file 2 — REPORTING SUMMARY [file 41537_2022_213_MOESM2_ESM.pdf]

nature portfolio

Corresponding author(s): Sung-Wan Kim

Last updated by author(s): Dec 3, 2021

Reporting Summary

Nature Portfolio wishes to improve the reproducibility of the work that we publish. This form provides structure for consistency and transparency in reporting. For further information on Nature Portfolio policies, see our Editorial Policies and the Editorial Policy Checklist.

Statistics

For all statistical analyses, confirm that the following items are present in the figure legend, table legend, main text, or Methods section.

n/a	Confirmed	
☐	☒	The exact sample size (n) for each experimental group/condition, given as a discrete number and unit of measurement
☐	☒	A statement on whether measurements were taken from distinct samples or whether the same sample was measured repeatedly
☐	☒	The statistical test(s) used AND whether they are one- or two-sided<br>*Only common tests should be described solely by name; describe more complex techniques in the Methods section.*
☐	☒	A description of all covariates tested
☐	☒	A description of any assumptions or corrections, such as tests of normality and adjustment for multiple comparisons
☐	☒	A full description of the statistical parameters including central tendency (e.g. means) or other basic estimates (e.g. regression coefficient) AND variation (e.g. standard deviation) or associated estimates of uncertainty (e.g. confidence intervals)
☐	☒	For null hypothesis testing, the test statistic (e.g. F, t, r) with confidence intervals, effect sizes, degrees of freedom and P value noted<br>*Give P values as exact values whenever suitable.*
☐	☒	For Bayesian analysis, information on the choice of priors and Markov chain Monte Carlo settings
☐	☒	For hierarchical and complex designs, identification of the appropriate level for tests and full reporting of outcomes
☐	☒	Estimates of effect sizes (e.g. Cohen's d, Pearson's r), indicating how they were calculated

Our web collection on statistics for biologists contains articles on many of the points above.

Software and code

Policy information about availability of computer code

Data collection	no software was used.
Data analysis	no software was used.

For manuscripts utilizing custom algorithms or software that are central to the research but not yet described in published literature, software must be made available to editors and reviewers. We strongly encourage code deposition in a community repository (e.g. GitHub). See the Nature Portfolio guidelines for submitting code & software for further information.

Data

Policy information about availability of data

All manuscripts must include a data availability statement. This statement should provide the following information, where applicable:

- Accession codes, unique identifiers, or web links for publicly available datasets
- A description of any restrictions on data availability
- For clinical datasets or third party data, please ensure that the statement adheres to our policy

The data that provide the findings of this study are available from the corresponding author upon reasonable request.

March 2021

Field-specific reporting

Please select the one below that is the best fit for your research. If you are not sure, read the appropriate sections before making your selection.

☐ Life sciences ☒ Behavioural & social sciences ☐ Ecological, evolutionary & environmental sciences

For a reference copy of the document with all sections, see nature.com/documents/nr-reporting-summary-flat.pdf

Behavioural & social sciences study design

All studies must disclose on these points even when the disclosure is negative.

Study description	Quantitative cross-sectional study
Research sample	We conducted an anonymous online survey of residents aged 19–65 years from three metropolitan areas according to the prevalence of COVID-19 using a quota sampling method considering age and sex. The online survey was conducted via a service provider (Macromill Embrain) in which all subjects were panelists. We included patients aged 19–65 years who were being treated for a schizophrenia spectrum disorder in a community mental health center or psychiatric outpatient clinic.
Sampling strategy	Participants of the general population were recruited using a quota sampling method considering age and sex. In this study, the model fit was verified using CFI, TLI, and RMSEA, which are not sensitive to sample size, consider the simplicity of the model, and have established criteria for evaluating the fit. The fit of the study model was satisfactory for all indices except χ2 (Figure 1, Table 4).
Data collection	Data collections were conducted via a paper-and-pencil survey for patients with a schizophrenia spectrum disorder and via an anonymous online survey for the general population. The researcher was blind to the study hypothesis during data collection.
Timing	Data collections were conducted between April and July 2020.
Data exclusions	No final data were excluded from the analyses.
Non-participation	A total of 535 were dropped out from the data collection for the general population because they responded with the same answer option throughout, responded too quickly, or for other reasons.
Randomization	non-applicable

Reporting for specific materials, systems and methods

We require information from authors about some types of materials, experimental systems and methods used in many studies. Here, indicate whether each material, system or method listed is relevant to your study. If you are not sure if a list item applies to your research, read the appropriate section before selecting a response.

Materials & experimental systems

n/a	Involved in the study	
☒	☐	Antibodies
☒	☐	Eukaryotic cell lines
☒	☐	Palaeontology and archaeology
☒	☐	Animals and other organisms
☐	☒	Human research participants
☒	☐	Clinical data
☒	☐	Dual use research of concern

Methods

n/a	Involved in the study	
☒	☐	ChIP-seq
☒	☐	Flow cytometry
☒	☐	MRI-based neuroimaging

Human research participants

Policy information about studies involving human research participants

Population characteristics	See above.
Recruitment	See above.
Ethics oversight	The study was approved by the Chonnam National University Hospital Institutional Review Board (CNUH-2020-092).

Note that full information on the approval of the study protocol must also be provided in the manuscript.
